# Proteomic Analysis of Mice Fed Methionine and Choline Deficient Diet Reveals Marker Proteins Associated with Steatohepatitis

**DOI:** 10.1371/journal.pone.0120577

**Published:** 2015-04-07

**Authors:** Su Jin Lee, Jeong Han Kang, Waqas Iqbal, Oh-Shin Kwon

**Affiliations:** 1 School of Life Science, College of Natural Science, Kyungpook National University, Daegu, Korea; 2 Thoracic Disease Research Unit, Department of Biochemistry and Molecular Biology and Mayo Clinic Cancer Center, Mayo Clinic College of Medicine, Rochester, Minnesota, United States of America; Centro de Investigación en Medicina Aplicada (CIMA), SPAIN

## Abstract

The mechanisms underlying the progression of simple steatosis to steatohepatitis are yet to be elucidated. To identify the proteins involved in the development of liver tissue inflammation, we performed comparative proteomic analysis of non-alcoholic steatohepatitis (NASH). Mice fed a methionine and choline deficient diet (MCD) developed hepatic steatosis characterized by increased free fatty acid (FFA) and triglyceride levels as well as alpha-SMA. Two-dimensional proteomic analysis revealed that the change from the normal diet to the MCD diet affected the expressions of 50 proteins. The most-pronounced changes were observed in the expression of proteins involved in Met metabolism and oxidative stress, most of which were significantly downregulated in NASH model animals. Peroxiredoxin (Prx) is the most interesting among the modulated proteins identified in this study. In particular, cross-regulated Prx1 and Prx6 are likely to participate in cellular defense against the development of hepatitis. Thus, these Prx isoforms may be a useful new marker for early stage steatohepatitis. Moreover, curcumin treatment results in alleviation of the severity of hepatic inflammation in steatohepatitis. Notably, curcumin administration in MCD-fed mice dramatically reduced CYP2E1 as well as Prx1 expression, while upregulating Prx6 expression. These findings suggest that curcumin may have a protective role against MCD fed-induced oxidative stress.

## Introduction

Fatty liver disease is commonly associated with metabolic disorder or alcohol abuse and is characterized by the accumulation of fat in the liver. Non-alcoholic fatty liver disease (NAFLD) is frequently associated with elements of the metabolic syndrome, namely obesity, type 2 diabetes, and dyslipidemia. The pathophysiological picture of NAFLD ranges between simple steatosis, non-alcoholic steatohepatitis (NASH), and progressive fibrosis [1]. The mechanisms of simple steatosis progression to NASH are not yet fully understood. One proposed model for the development steatohepatitis is based upon the “two-hit” theory [2]. The first hit being the generation of a fatty liver (steatosis), and consequent hepatic insulin resistance. The second hit entails oxidative stress, which leads to up-regulation of proinflammatory cytokines. The exact reason oxidative stress occurs in hepatitis patients is still unclear. However, it is now well established that an intracellular accumulation of excess free fatty acids (FFA) is associated with cellular dysfunction in a condition known as lipotoxicity, which triggers inflammation and tissue repair in the form of fibrosis[3],[4].

Reactive oxygen species (ROS) mediated lipid peroxidation may be strongly associated with the pathogenesis of NASH. Oxidative stress can be attributed to the presence of aldehyde dehydrogenase (ALDH), catalase, and cytochrome P450 2E1 (CYP2E1) [5]. In particular, the microsomal enzyme CYP2E1 is a major contributor to ethanol-induced ROS production in the liver as well as in NASH. Accumulation of ROS tends to induce lipid peroxidation of cellular membranes, resulting in hepatocyte injury [6], [7]. Depletion of anti-oxidant defenses is also believed to be the cause of oxidative stress [8]. However, little is still known about the role of anti-oxidant defense systems in the development of oxidative stress in NASH. Thioredoxin (Trx) and glutathione (GSH) are two major thiol-dependent antioxidant systems in mammalian cells. Trx participates in the redox regulation of NO signaling whereas the GSH system plays an important role in cellular defense against oxidative stress via the efficient removal of various ROS by glutathione peroxidase (GPx)[9]. In addition to these, catalase, superoxide dismutase (SOD), and peroxiredoxins (Prx) are also important antioxidant enzymes in the defense against oxidative stress [10]. Catalase, cytoplasmic GPx1, and Prx are mainly involved in the decomposition process of hydrogen peroxide (H_2_O_2_). Prx proteins are a class of thiol-dependent antioxidant protein and are present in all biological kingdoms from bacteria to mammals, and have received considerable attention in recent years due to their role in regulating cellular signaling pathways. Prx isoforms show distinct intracellular distributions. They are localized in the cytosol, mitochondria, endoplasmic reticulum (ER), and peroxisome [11].

Curcumin is the prominent yellow pigment in turmeric, a widely used spice and food coloring agent with anti-inflammatory and anti-cancer properties [12]. Moreover, curcumin chelates and scavenges ROS, and induces anti-oxidant enzymes [13],[14]. Diet-induced obesity is widely reported to be suppressed by curcumin [15]. There is also evidence that curcumin treatment may help protect against liver injury caused by various factors, including carbon tetrachloride and ethanol [16]. It has been demonstrated to prevent several pathologies related to oxidative damage such as UV irradiation-induced oxidative stress, inorganic arsenic-induced hepatotoxicity and methylglyoxal-induced oxidative stress [17–19]. However, the hepatoprotective effects and molecular mechanism of curcumin action on acute liver injury are still unknown.

To increase our understanding of the mechanism that links steatosis to hepatitis, animal models of NASH are particularly useful. To date, however, no animal models adequately reproduce the pathogenetic mechanisms and histological features observed in human cases of NASH. Nevertheless, the administration of a methionine and choline deficient (MCD) diet to rodents has been proven to be a useful experimental model for this disease [20], [21]. The MCD rodent model shows clear evidence of increased oxidative stress [22], [23], which seems to be important in the progression from steatosis to steatohepatitis. Proteomics offers a unique tool to provide important clues to the mechanisms involved in this complex process. However, very little research using proteomics to study MCD diet-induced steatohepatitis has been reported. Thus, we initiated the characterization of changes in the liver proteome during the progression of steatohepatitis. Here, we report a search for proteins that are specifically up or downregulated in MCD-induced mice as compared to control animals. In addition, we also describe the effects of curcumin on steatohepatitis induced by the MCD diet, with particular attention to the involvement of Prx isoforms in inflammation and liver injury. Our findings suggest that Prx isoforms are promising candidates for a NASH biomarker.

## Materials and Methods

### 1. Ethics statements

This study was carried out in strict accordance with the recommendations in the Guide for the Care and Use of Laboratory Animals of the National Institutes of Health. Animal studies including the issue of ethical treatment of the animals were all reviewed and approved by Animal Ethics Committee of Kyungpook National University, and the protocols were approved by this committee. All surgery was performed under ketamine anesthesia, and all efforts were made to minimize suffering.

### 2. Animals

Male C57BL/6J mice were obtained from Orient Bio, Inc. (Seoul, Korea). All animals were housed in a temperature-controlled (20–24°C) and humidity-controlled (45–55%) environment with a 12: 12-h light: dark cycle. The groups were as follows: (1) Normal diet (ND) (n = 10): animals fed the standard diet for 12 weeks; (2) MCD (n = 8): animals fed the MCD diet for 3 weeks (S1 Table); and (3) MCD diet supplemented with curcumin (n = 8): animals treated with MCD + curcumin diet for 3 weeks.

### 3. Two-dimensional gel electrophoresis

Two-dimensional gel electrophoresis (2DE) was performed using an established procedure [24]. Briefly, whole liver tissue lysate (400 μg) was added to a 24 cm immobilized pH 4–7 linear gradient strip (Amersham Biosciences), which was rehydrated in an Ettan IPGphor isoelectric focusing (IEF) system. Complete sample uptake into the strip was achieved after 12 h at 20°C. The focusing was as follows: 500 V (1 h), 1000 V (1 h), 4000 V (2 h), and 8000 V (6 h). For the second dimension of electrophoresis, the IPG strip was incubated in equilibration buffer (125 mM Tris [pH 6.8] containing 6 M urea, 30% glycerol, and 2% SDS) containing 65 mM DTT for 15 min, and then incubated for 15 min in equilibration buffer supplemented with 2.5% (w/v) iodoacetamide. The equilibrated IPG strip was transferred onto a 12% SDS gel for SDS-PAGE and run at about 120 V using Ettan DALTsix Larger Vertical System (Amersham Biosciences). Gel staining was performed as described by Kang, et al. [25]. To ensure the reproducibility of the observed changes in protein expression, experiments were performed that made two groups, which is three pools of three independent liver samples of ND- or MCD-fed mice. Each group was run in duplicate. For the differential analysis, statistical significance was estimated with the Student’s *t* test. Values of *p* < 0.05 were considered significant.

### 4. In-gel digestion & MALDI-TOF analysis

Protein spots were in-gel digested using trypsin and samples were prepared for MALDI-TOF analysis. In short, selected gel pieces were washed for 10 min (25 mM NH_4_HCO_3_/50% acetonitrile), vacuum dried, and digested overnight (50 mM NH_4_HCO/20 μg/mL trypsin) at 37°C. The supernatant was transferred to a fresh tube and dried in a vacuum centrifuge. The dried extract was dissolved in 0.5% trifluoroacetic acid (TFA) and concentrated and desalted using C_18_ZipTip (Millipore, Billerica, MA, USA) according to the manufacturer’s protocol prior to spotting on the steel MALDI plate. For MALDI-TOF analysis, a 4700 proteomics analyzer (Applied Biosystems, Foster City, CA, USA) was used with reflector/delayed mode. Data were analyzed using GPS Explorer software (Applied Biosystems) and MASCOT software (Matrix Science, London, UK) using mouse protein sequences in the NCBI non-redundant database as a reference set. Search parameters were a precursor mass tolerance of 30 ppm for MS and 0.7 Da for MS/MS was allowed. In addition, up to two missed cleavage were allowed; oxidation of methionine was set as variable modification and carboxyamidomethylation of cysteine as fixed modification.

### 5. Western blot

Cell lysates (25 μg of protein) were separated by 12% SDS-PAGE, transferred to PVDF membranes (Millipore, Billerica, MA, USA), blocked with 5% nonfat dry milk, and probed with antibodies at 4°C overnight. Antibodies against α-SMA (1:1000, Epitomics, Burlingame, CA, USA), CYP2E1 (1:1000, Cell signaling Technology Inc., Beverly, MA, USA) and Prx1 and 6 (1:1000, Abcam, Cambridge, MA, USA) were used as probes. The anti-GAPDH antibody (1:1000, Santa Cruz, CA, USA) was used as a loading control.

### 6. Immunohistochemistry

For histological analysis, liver sections were stained with hematoxylin-eosin (H&E). The primary antibodies applied to the tissue sections were a rat antibody against F4/80 (dilution 1:500; Serotec, Oxford, UK), a mouse antibody against α-SMA (1:200), 4-HNE (1:200, Millipore, Billerica, MA, USA), CYP2E1 (1:200), and Prx1 and Prx6 (1:200). Peroxidase activity was revealed using DAB substrate, slides were counterstained with Hematoxylin (Sigma Chemical, St. Louis, MO, USA), then dehydrated and mounted in Safemount embedding medium (Labonord, France).

### 7. Statistical Analysis

All values are represented as means ± SD of the indicated number of measurements. A one-way analysis of variance test was used to determine significance, requiring * *p* < 0.05 for statistical significance.

## Results

### 1. Hepatic lipid deposition in mice fed MCD

**Fig 1:**
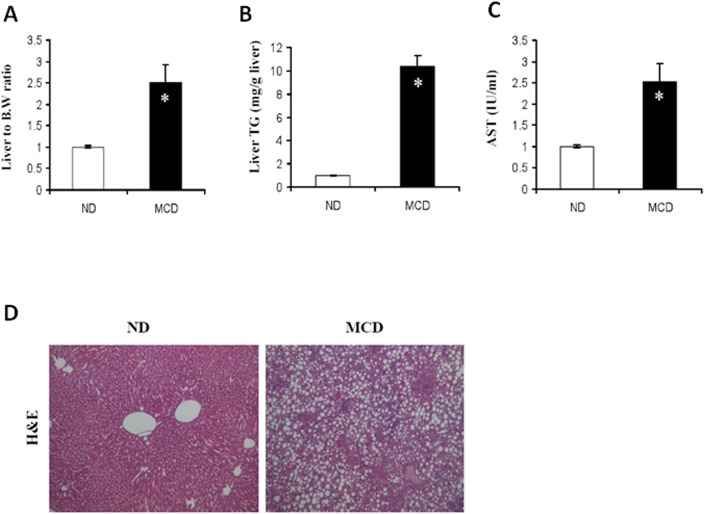
Effect of MCD diet on weight loss and hepatocellular damage. (A) Ratio of liver to body weight, (B) Hepatic triglyceride, and (C) AST values were analyzed in mice fed the ND or MCD diet. A significant increase in serum AST levels was observed in MCD-fed mice. Mean ± SD of results from ND (n = 5), MCD (n = 10). (D) The histology of liver sections from a ND, MCD by Hematoxylin-eosin (H&E) staining (original magnification 200×).

We initially placed C57BL/6 mice on an MCD diet, which has been extensively shown to be associated with progressive fibrosing steatohepatitis, pathologically similar to human steatohepatitis. We observed significant hepatic fat accumulation induced by MCD feeding. A significant increase in the liver-to-body weight ratio was observed in the MCD group (Fig. 1A). The total hepatic triglyceride content and serum AST of the MCD fed mice was significantly greater than that of ND mice (Fig. 1B and 1C respectively). For closer observation of liver changes, histology sections were stained with H&E and examined by light microscopy (Fig. 1D), revealing macrovesicular lipid accumulation.

### 2. Hepatic inflammation and Kupffer cell activation

**Fig 2:**
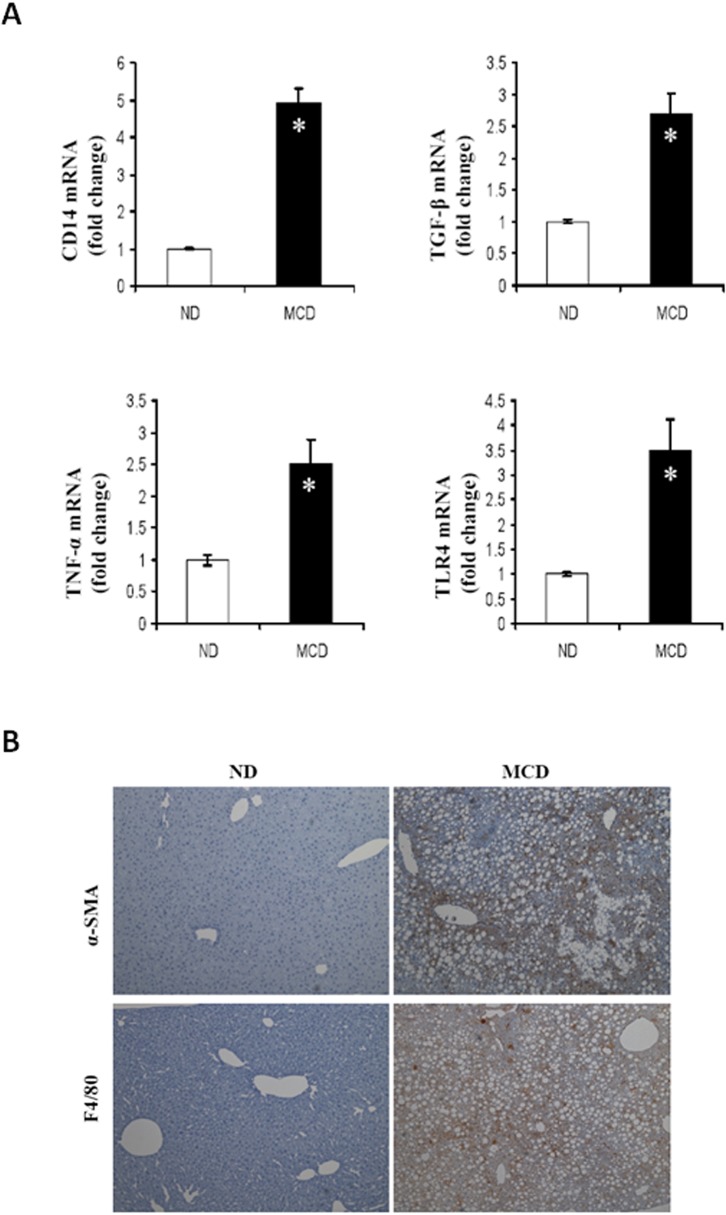
Expression levels of proteins functioning in the development of steatosis and steatohepatitis. Liver tissue extracts prepared from mice fed ND or MCD. (A) Hepatic levels of *CD14*, *TNF-α*, *TGFβ1 and TLR4* mRNA were measured by qRT-PCR. Genes were normalized to *GAPDH* RNA as an internal standard, and data are shown as fold increase. *P < 0.05. (B) Representative photomicrographs of α-SMA and F4/80 immunostaining in the liver of ND, MCD fed mice.

In order to explore MCD diet-dependent profibrogenic effects, we performed qRT-PCR analysis of liver homogenates (Fig. 2A, primers of qRT-PCR; S2 Table). The co-receptor, *CD14* in the TLR4 receptor complex significantly increased as determined by mRNA levels in the MCD fed mice. In addition, genes associated with fibrosis and cytokines, such as *TGFβ1*, *TNFα*, and *TLR4* were also induced in the livers of MCD fed mice. Liver changes in MCD-fed mice were associated with a marked increase in inflammation. To evaluate the effect of MCD feeding on hepatic stellate cells (HSCs) and macrophage activation *in vivo*, α-SMA and F4/80 positive cells were examined, respectively, in liver tissues using immunohistochemistry (Fig. 2B). The levels of α-SMA and F4/80 were significantly increased in the MCD-fed group as compared to ND-fed mice. α-SMA is a unique marker for activated stellate cells and myofibroblasts, whereas F4/80 is a macrophage marker.

### 3. Proteomics analysis

**Fig 3:**
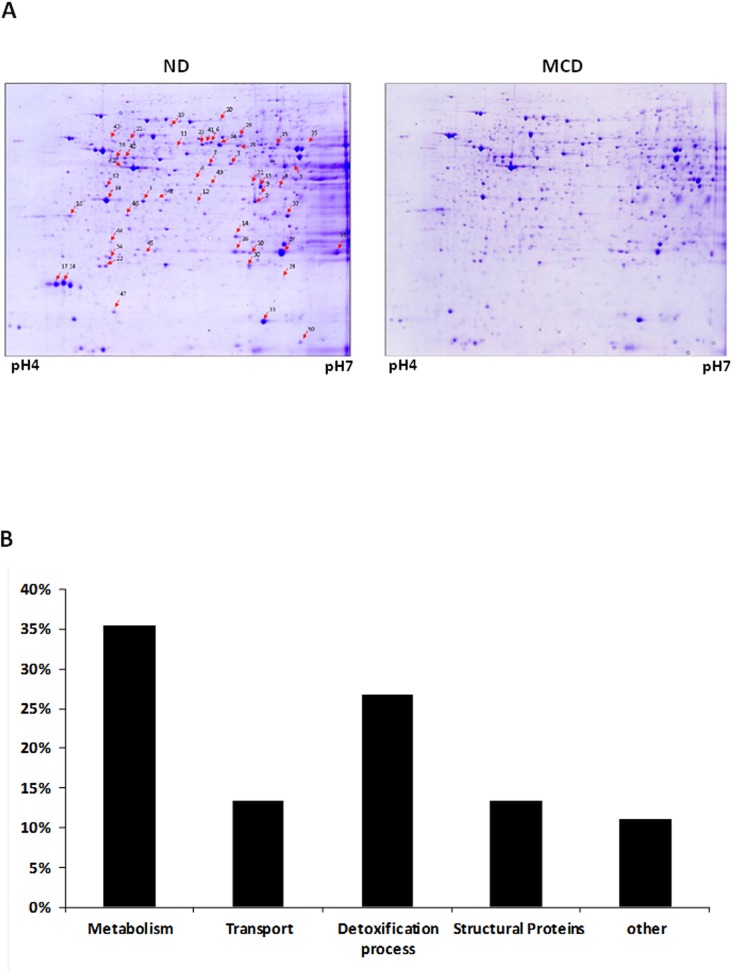
Protein expression map of mouse liver and Classification of the proteins. (A) Coomassie stained 2DE gel shows proteins derived from mice fed ND or MCD. Proteins from mouse liver were loaded on a 24 IPG strip (pH 4–7) and then run on an SDS-PAGE (12%). Protein spots noticeably affected by MCD feeding are indicated by arrows. The numbers on the gels correspond to spot numbers in Table 1. (B) Bar graph representing the distribution of the identified proteins according to biological function. Assignments were made based on information from the NCBI (www.ncbi.nlm.nih.gov/PubMed) and the Swiss-Prot/TrEMBL protein knowledgebase (http://au.expasy.org/sport) websites.

Differential proteomic analysis of the soluble protein fraction of the sampled liver tissues yielded several significant changes in MCD-fed mice. Fig. 2 shows typical 2-DE patterns of mouse liver. Gel images for the mice livers were analyzed with Image Master Platinum 7.0 software. Protein spots significantly affected by MCD feeding are marked by arrows with numbers (Fig. 3A). Protein spots from preparative gels were subjected to in-gel tryptic digestion and MALDI–TOF analysis. As shown in Fig. 3B, the differentially expressed proteins are grouped and classified according to their biological function. A majority of differentially expressed proteins are related to metabolism, detoxification/oxidative stress, and homeostasis. These results suggest that the differentially expressed proteins are involved in the disturbance of metabolic homeostasis and hepatocellular oxidative stress in steatohepatitis. Table 1 lists the most strongly differentially expressed proteins, which exhibit at least a 1.5 fold difference in expression. The numbers indicated on the gels correspond to the numbering given in Table 1. It is noteworthy that 12 proteins are involved in detoxification processes, and 9 of these have reduced expression in MCD fed mice.

**Table 1:** List of identified proteins.

No^a^	Identified protein	Accession No.	MW (KDa)	PI	MOWSE Score^b^	coverage (%)^c^	Fold change
**Metabolism**							
1	Ornithine aminotransferase, mitochondrial	P29758	48.4	6.2	2.15e+9	55.1	0.4
2	Malate dehydrogenase, cytoplasmic (MDHC)	P14152	36.5	6.2	1304	23.4	0.5
3	Inorganic pyrophosphatase	Q9D819	32.7	5.4	58907	41.9	2.3
4	Aspartate aminotransferase, cytoplasmic	P05201	46.2	6.7	100196	17.7	1.7
5	Acyl-coenzyme A thioesterase 2, mitochondrial	Q9QYR9	49.7	6.0	2.82e+7	26.5	3.2
6	Formimidoyltransferase-cyclodeaminase	Q91XD4	58.9	5.8	83282	30.9	0.18
7	Adenosine kinase	P55264	40.1	5.8	1.05e+6	37.1	0.55
8	Fructose-1,6-bisphosphatase 1	Q9QXD6	36.9	6.1	1.23e+6	44.7	2.4
9	PCTP-like protein	Q9JMD3	33.0	6.7	36911	33.3	0.6
10	Anhydrolase domain-containing protein 14B	Q8VCR7	22.5	5.8	9169	42.4	1.64
11	Glutathione synthetase	P51855	52.2	5.6	73657	24.1	2.5
12	Ketohexokinase	P97328	32.8	5.8	2322	25.8	2.2
13	Pyruvate dehydrogenase E1 component subunit beta, mitochondrial	Q9D051	38.9	6.4	1.05e+6	41.8	1.8
14	IEMT (Indolethylamine *N*-methyltransferase)	P40936	29.5	6.0	5.81e+6	42.8	0.3
15	Isocitrate dehydrogenase [NAD] subunit alpha, mitochondrial	Q9D6R2	39.6	6.3	104	10.9	0.4
16	Annexin A5	P48036	35.8	4.8	22728	7.3	2.2
**Transport**							
17/18	Major urinary proteins 11 and 8 (Fragment)	P04938	17.6	4.9	617597	66.2	0.1
19/20	Serum albumin	P07724	68.7	5.7	1.39e+10	41.0	1.9
21	Vitamin D-binding protein	P21614	53.6	5.4	2.19e+6	29.8	0.6
22	Phosphatidylethanolamine-binding protein 1	P70296	20.8	5.2	2448	25.8	0.58
23/24	Selenium-binding protein 2	Q63836	52.6	5.8	2.83e+11	53.2	0.13
25	Selenium-binding protein 1	P17563	52.5	5.9	1.78e+13	69.1	0.6
**Oxidative Stress /Detoxification processes**							
26/27	AOP2 (Peroxiredoxin-6)	O08709	26.3	6.2	2350	18.5	0.47
28	Peroxiredoxin-1	Q9D6L8	18.1	6.3	282	27.3	3.5
29	Epoxide hydrolase 2	P34914	62.5	5.9	1.32e+11	48.2	0.37
30	Glutathione peroxidase 1 (GPx)	P11352	22.3	6.7	130647	41.8	0.55
31	Trans-1,2-dihydrobenzene-1,2-diol dehydrogenase/Dimeric dihydrodiol dehydrogenase	Q9DBB8	36.3	6.0	13018	27.0	0.6
32	Sulfotransferase family cytosolic 2B member 1	O35400	38.3	5.0	1192	15.4	2.6
33	SODC (Superoxide dismutase [Cu-Zn])	P08228	15.9	6.0	1996	41.6	0.5
34	Lactoylglutathione lyase	Q9CPU0	20.8	5.2	2.59e+6	62.5	0.55
35	Aldehyde dehydrogenase X, mitochondrial	Q9CZS1	57.6	6.6	2.34e+7	33.5	4.5
36	Glutathione S-transferase P1 (GST)	P19157	23.6	7.7	4.83e+6	42.4	0.33
37	3-hydroxyanthranilate 3,4-dioxygenase	Q78JT3	32.8	6.1	9.05e+9	74.5	0.3
38	Regucalcin	Q64374	33.4	5.2	1.31e+7	53.5	0.5
**Structural Proteins**							
39/40	Keratin, type I cytoskeletal 18	P05784	47.5	5.2	2.73e+6	32.2	2.3
41	Keratin, type II cytoskeletal 8	P11679	54.6	5.2	20500	25.7	1.8
42	Actin-related protein T1	Q9D9J3	42.2	5.2	107	16.8	2
43	Vimentin	P20152	53.7	5.1	178	15.5	2.5
44	Rho GDP-dissociation inhibitor 1	Q99PT1	23.4	5.1	2545	41.7	1.8
45	Regulator of G-protein signaling 20	Q9QZB1	27.0	5.1	22	12.1	0.62
**Others**							
46	Phenazine biosynthesis like domain containing protein 2	Q9CXN7	33.4	5.2	3.83e+6	17.1	0.41
47	Eukaryotic translation initiation factor 5A-1	Q6EWQ7	16.8	5.1	3706	40.3	0.15
48	Putative deoxyribonuclease TATDN1	Q6P8M1	33.4	5.8	4026	21.4	1.85
49	60S acidic ribosomal protein P0	P14869	34.2	5.9	1.21e+7	60.6	0.52
50	Histidine triad nucleotide-binding protein 1	P70349	13.8	6.4	15430	57.9	0.63

Spot ID numbers correspond to the Spot numbers on the gel.

a. Spot numbering as shown in 2-DE gel in Fig. 3.

b. Protein score (based on combined and mass/mass spectrum).

c. Sequence coverage identified from MS/MS data

### 4. Validations of selected proteins including oxidative stress and antioxidant proteins

**Fig 4:**
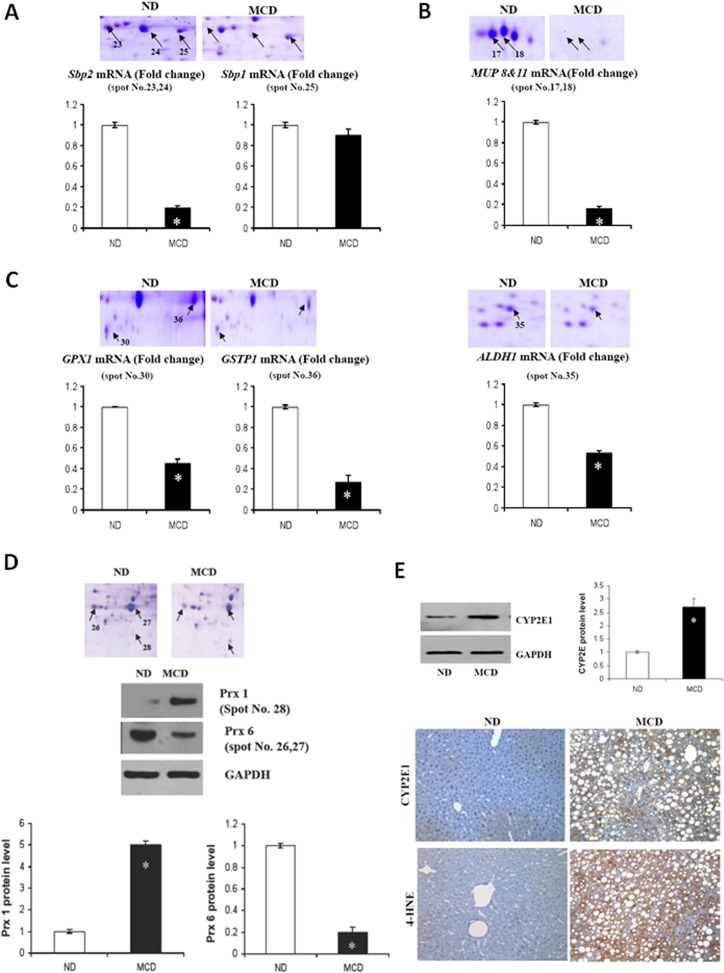
Validations of selected proteins including antioxidant protein. Liver tissue extracts were prepared from mice fed the MCD or the ND. Representative sections of 2-D gels are amplified to depict some of the identified proteins. The relative expression levels of mRNA were determined by qRT-PCR analysis. Commonly, liver extracts from a pool of 3 mice in each group were used. Genes were normalized to GAPDH RNA as an internal standard, and data are shown as fold change. *P < 0.05. (A) Upper panel shows hepatic expression levels of Sbp 2 (spot 23 & 24) in the ND and MCD mice. Sbp1 (spot 25) was used as a negative control. Lower panel shows the relative expression level of mRNA determined by qRT-PCR analysis. (B) Hepatic expression profiles of MUPs (spot 17 & 18) and mRNA compared to each other. (C) The expression levels of GSTP1, GPX1, and ALDH1 in MCD compared to ND. Levels of *GSTP1*, *GPX1*, and *ALDH1* mRNAs as measured by qRT-PCR. (D) Enlarged areas of gels derived from ND and MCD mice show the expression profiles of PRX 1 and 6. Differential expression levels of PRx1 and 6 in the liver tissue were confirmed by Western blot analysis. Data are representative of three independent experiments. (E) CYP2E1 protein content was quantified by Western blot, using equal quantities of total liver protein. For statistical significance, three liver extracts from each individual were used for each group. Expression levels were normalized relative to GAPDH. Data are representative of three independent experiments. Liver sections from ND- and MCD-fed mice were subjected to immunohistochemical analysis with antibodies to CYP2E1 and 4-HNE.

In order to determine whether the changes in protein expression are consistent with changes in gene expression, mRNA contents were compared with the results from 2-DE. In particular, we observed that Sbp2 (spot 23 and 24) was dramatically downregulated in protein expression in MCD fed mice (Fig. 4A). Consistently, a decrease in *Sbp2* mRNA took place in a parallel manner. These results confirmed that Sbp2 is transcriptionally downregulated in steatohepatitis. In contrast to Sbp2, Sbp1 protein (spot 25) and mRNA levels were not changed. In addition, major urinary protein (MUP), also known as lipocalin, showed the most significant decrease in the MCD fed mice as determined by 2DE analysis (Fig. 4B).

Proteins involved in cellular defensive strategies against oxidative stress also showed coordinated changes in their expression levels. For instance, proteomic and qPCR analyses showed decreased expression of antioxidant molecules such as glutathione peroxidase 1 (GPx1), GSTP1, and ALDH1 in MCD-fed mice (Fig. 4C). In addition, we investigated the effect of MCD fed mice on the expression of Prxs in the liver of mice by western blotting (Fig. 4D). While hepatic Prx1 expression levels significantly increased in MCD fed mice, those of Prx6 dramatically decreased. Induction of CYP2E1 is a central pathway in generating oxidative stress and releasing ROS. Western blot assays showed a dramatic increase in the expression of CYP2E1 in MCD-fed mice as compared to ND-fed mice (Fig. 4E). The western blot results of CYP2E1 were confirmed by immunohistochemistry. Moreover, 4-HNE adduct formation was rare in ND fed mice but was prominent in MCD fed mice. The 4-HNE formation indicates lipid peroxidation.

### 5. Curcumin prevents oxidative stress and recovers antioxidant protein expression in mice fed MCD

**Fig 5:**
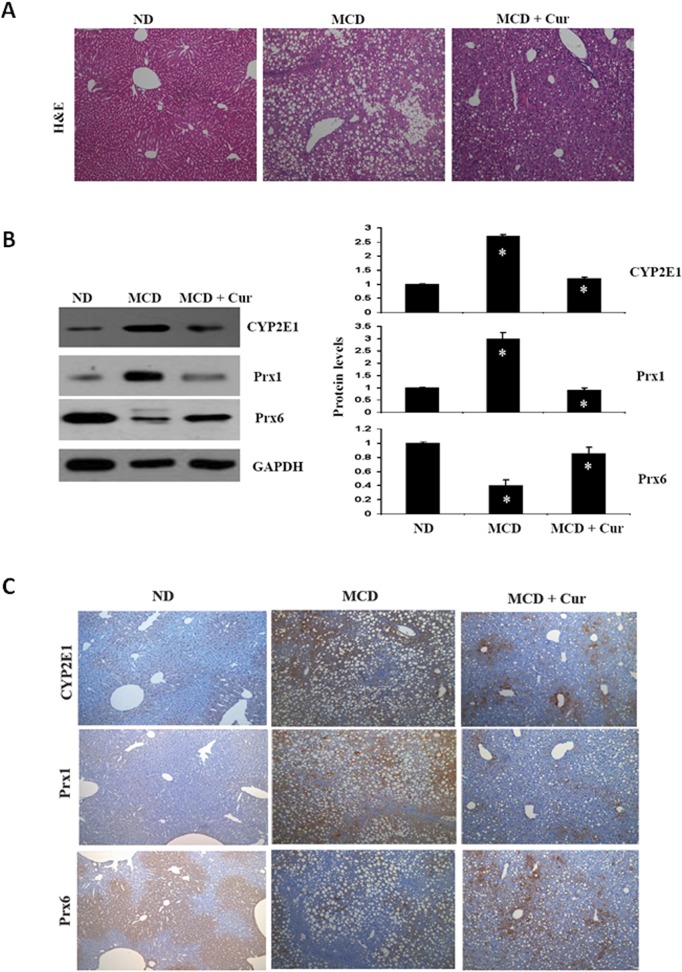
Curcumin prevents oxidative stress and recovers antioxidant protein expression in mice fed MCD. (A) Effect of curcumin on hepatic morphology in mice fed the control diet, the MCD diet and MCD + curcumin diet. The histology of ND and MCD liver sections by Hematoxylin-eosin (H&E) staining (original magnification 200×). (B) and (C) The effect of curcumin on CYP2E1, Prx1, and Prx6 protein levels in curcumin-supplemented diet. CYP2E1, Prx1 and Prx6 protein levels were determined by Western blot and immunohistochemical analysis of livers of mice fed the control or MCD diet alone or MCD + curcumin diet. Data are mean ± SD (n = 6/group). *P < 0.05

In that curcumin is a potential antioxidant and anti-inflammatory agent [26], we investigated the effects of curcumin on the expression of antioxidant proteins in MCD-fed mice. As shown in Fig. 5A, curcumin led to a significant improvement of the liver pathology induced by MCD feeding. Further, the curcumin-treated mice displayed significantly reduced CYP2E1 and Prx1 expression levels, while Prx6 protein expression increased (Fig. 5B). These western blot results were confirmed by immunohistochemistry analysis as shown in Fig. 5C. Hepatic CYP2E1 and Prx1 expression levels in MCD fed mice were upregulated, whereas Prx 6 was downregulated

## Discussion

NASH is characterized by excessive fat accumulation in the liver with attendant cell injury and inflammation. The cause of progression of hepatosteatosis to NASH is still poorly understood. The goal of this work was to identify differentially expressed proteins in steatohepatitis, and to characterize metabolic pathways that may explain the onset of steatohepatitis. In this study, a proteomic approach was used to determine the effects of feeding mice the MCD diet as compared to the ND diet. This resulted in our identifying ∼50 differentially expressed proteins; a large number of which being related to oxidative stress and cellular defense against reactive oxygen radicals.

Reactive oxygen species (ROS) are continually generated during metabolism and subsequently scavenged by antioxidant systems. Perturbation of this equilibrium leads to oxidative stress. Therefore, the final mediator of the development of steatohepatitis seems to be oxidative stress due to production of ROS with decreased antioxidant defenses. Our results demonstrate a significant increase of hepatic CYP2E1 in the MCD fed mice as determined by immunohistochemistry and Western analysis. Indeed, the involvement of CYP2E1 in NASH has been established in MCD-fed mice, where liver injury and fibrosis were associated with induction of CYP2E1. Conversely, Cyp2e1−/− mice were protected from this damage [22]. Increased generation of reactive oxygen species (ROS) and other reactive intermediates and/or decreased efficiency of antioxidant defenses actively contribute to parenchymal cell death and excess tissue remodeling and fibrogenesis [27],[28]. On the other hand, CYP2E1 is also a major contributor to ethanol-induced ROS production in the liver. CYP2E1 metabolizes ethanol to acetaldehyde, and increases oxidative stress by the production of ROS and lipid peroxides.

Lipid peroxidation seems to represent an important mechanism that initiates and perpetuates inflammation. Lipid peroxidation products have been referred to as surrogate indicators for oxidative stress. 4-Hydroxy-2,3-(E)-nonenal (4-HNE), a major aldehyde product of lipid peroxidation, is metabolized through oxidative (aldehyde dehydrogenase), reductive (alcohol dehydrogenase), and conjugative (glutathione S-transferase; GST) pathways [29]. In particular, the aldehyde dehydrogenase (ALDH) family is important in cellular defense against exogenous toxic aldehydes and endogenous aldehydes, such as those derived from lipid peroxidation [30]. In this study, ALDH1 was downregulated in MCD-fed mice as compared to ND mice. Other research groups have reported that ADLH was down regulated in an alcoholic liver disease model [31]. Since an increased level of ROS is commonly accompanied by fat accumulation, we can predict a reduction of ALDH expression in liver tissue following oxidative stress.

Detoxification of reactive oxygen and reactive nitrogen species is a standard cellular response to oxidative stress [32]. The first line of defense against free radicals is provided by SOD and GPx. GST is the second line of defense; its expression is regulated by cellular redox status. GPx, SOD, and GST expression was found to be downregulated in MCD-fed mice, leading to oxidative stress[33]. A decrease in the expression of GPx1 is likely to reflect an increase in toxic metabolite production, and can thus be regarded as a sign of developing oxidative stress. In addition, a down-regulation of SBP2 in mRNA and protein expression has been observed in this model of hepatitis. SBP2, a selenium transporter, is mainly expressed in the liver and predominantly incorporated into GPx. Under toxic conditions, such as alcohol abuse, the selenium level in liver is known to decrease [34]. Thus, SBP2 may be a useful marker in toxic-induced steatohepatitis. GSTP1 is a member of the GST family and plays an important role in detoxification by catalyzing the conjugation of many compounds with reduced glutathione. Expression of GSTP1 is associated with phase 1 detoxification of oxidative stress products. Recently, GSTP1 expression has been implicated in the regulation of cell proliferation and apoptosis through direct interaction with c-Jun N-terminal kinase (JNK) [35]. Thus, the proteome of steatohepatitis cells presented with a down-regulation of enzymes involved in the elimination of ROS and damaging metabolites, along with the depletion of cellular defensive reserves to withstand oxidative stress.

Prx proteins function in cellular protection against oxidative stress, regulation of cell proliferation, and modulation of intracellular signaling pathways that use hydrogen peroxide as a secondary signal transmitter. Further evidence for an altered redox potential in the liver is provided by the expression levels of Prx proteins. In this study, we showed that the expression levels of two Prx isoforms, namely Prx 1 and Prx 6, were significantly changed in MCD-fed mice. In addition, the expression pattern of all Prx isoforms in MCD-fed mice is considerably similar to those obtained from EtOH-fed mice (S1 Fig.), which may result in common pathogenic mechanisms of NAFLD and alcoholic liver disease. Prx 1 is a prime candidate for regulation of H_2_O_2_ signaling, initiated by cell-surface receptors. Moreover, Prx 1 is important in protecting against oxidative stress and inflammation, possibly through suppression of the NF-κB pathway [36]. We showed that expression level of Prx 1 is significantly upregulated in MCD-fed mice. An *in vivo* antioxidant function of Prx1 was recently demonstrated in the livers of ethanol-fed mice [37]. We also found that Prx4 was up regulated in both MCD- and EtOH-fed mice (S1 Fig.). We assume that changed Prx 4 expression may reflect a link between oxidative stress and the pathology of hepatitis. Recently, Rpdriguez-Suarez et al. have reported that Prx4 was up regulated in NAFLD patients [38]. Our results are consistent with these findings. Prx 4 is referred to as TRANK, a thioredoxin peroxidase related activator of NF-κB and c-Jun N-terminal kinase, and has been shown to function as a cytokine in the activation of NF-κB. On the other hand, Prx 6 reduces H_2_O_2_, short chain fatty acids, and phospholipids and thus protects against oxidative injury. Up-regulation of Prx 6 suppresses intracellular enzyme inactivation, membrane phospholipid peroxidation, and cell death, mediated by oxidative stress. In addition, Prx 6-deficient mice are susceptible to oxidative stress by hypoxia or paraquat treatment, leading to an increase in mortality and lung injury [39]. In the present study, we found a reduction in Prx 6 expression in MCD-fed mice, suggesting diminished capability to counteract oxidative stress in the liver. Likewise, Prx6 was significantly down-regulated in EtOH-fed rats [31]. Although the precise mechanism for gender susceptibility to alcoholic steatohepatitis is not well understood, Prx6 was found to lesser downregulated in females as compared to the males. It was also evident that the decrease in expression is likely due to increased degradation of this protein rather than though a decrease in synthesis and transcription [40]. Together, these changes of antioxidant proteins in expression are indicative of hepatocellular damage in increased ROS levels. We may conclude that Prx seems to emerge as the most-attractive candidate for a steatohepatitis biomarker. The presence of independent isoforms and high expression levels of Prx suggest its usefulness as an oxidative stress marker and the diagnosis of the liver disease.

The polyphenolic substance diferuloylmethane, commonly known as curcumin, is derived from the rhizomes of Curcuma longa and has been demonstrated to possess antioxidant activity *in vivo* [41], [42]. We investigated whether curcumin is capable of effectively alleviating the hepatic steatohepatitis induced by the MCD diet. Liver damage biomarkers, including AST and ALT, are increased in fatty liver disease mice, but reduced by curcumin supplementation. It has been reported that alcohol intake and a high-fat diet can promote the development of hepatic steatosis. Curcumin treatment is known to decrease triglyceride accumulation [15,43,44,] and suppress NF-kB activation via direct modifications on the NF-kB/IkB complex, and inhibition of IkB degradation [45]. In this report, we showed that curcumin prevents oxidative stress and significantly improves liver pathology induced in mice by MCD feeding. Hepatic CYP2E1 and Prx1 expression levels were significantly reduced in curcumin treatment mice, whereas Prx6 was dramatically increased. Therefore, although the regulatory mechanisms need to be further investigated, it is likely that curcumin plays a role in alleviating the severity of hepatic inflammation in steatohepatitis.

In summary, we have identified 50 differentially expressed proteins from the MCD-fed animal. As far as we are aware, the present work is the first proteomic analysis of MCD diet-induced NASH. Furthermore, our findings reveal an oxidative stress involving accumulation of hepatic toxicity. In particular, the defense proteins such as GPx1 and SBP2 were significantly down-regulated in steatohepatitis model. SBPs are believed to play a crucial role in the antitoxic function of selenite. Thus SBP2 substitution in toxic-induced NASH may yield a therapeutic strategy targeted against Sbp2 down-regulation. Among proteins involved with antioxidative stress, the Prx family was the most-pronounced change. Prx 1 and Prx 4 were strongly upregulated in MCD mice, whereas Prx 6 was downregulated. These results suggest a potentially important role for these proteins in hepatitis. We also found that curcumin treatment reverses changes in Prx expression and attenuates oxidative stress responses in MCD-feeding induced hepatitis. Consequently, Prx isoforms may serve as potential biomarkers, providing a new perspective on the pathogenesis of Prx in steatohepatitis.

## Supporting Information

S1 Fig Expression of Prx1 to 6 in mice fed MCD and EtOH. Liver tissue extracts were prepared from mice fed the MCD, EtOH and ND. Prx proteins content was quantified by Western blot, using equal quantities of total liver protein. Expression levels were normalized relative to GAPDH. EtOH-fed group: drank 5% wt/vol alcohol for 2 days, 20% wt/vol alcohol for 5 days, and 30% wt/vol alcohol for 6 week.
(DOC)

S1 Table Methionine and Choline Deficient (MCD) Diet Composition. (DOC)

S2 Table Primers used for real time PCR. (DOC)
