# Supplementary material for: Proteomic Analysis of Mice Fed Methionine and Choline Deficient Diet Reveals Marker Proteins Associated with Steatohepatitis
Source: PLoS One. 2015 Apr 7;10(4):e0120577. doi: 10.1371/journal.pone.0120577 (PMC4388516; doi:10.1371/journal.pone.0120577)
Supplement: S1 Fig — Liver tissue extracts were prepared from mice fed the MCD, EtOH and ND. Prx proteins content was quantified by Western blot, using equal quantities of total liver protein. Expression levels were normalized relative to GAPDH. EtOH-fed group: drank 5% wt/vol alcohol for 2 days, 20% wt/vol alcohol for 5 days, and 30% wt/vol alcohol for 6 week. (DOC) [file pone.0120577.s001.doc]

**
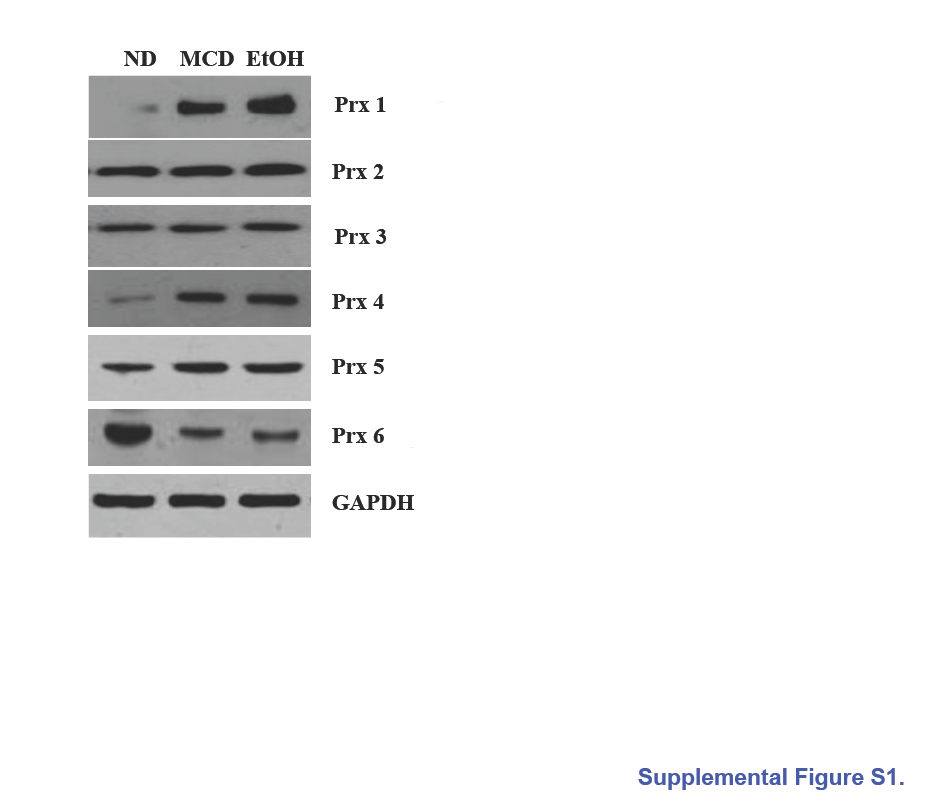
**

**Figure S1. Expression of Prx1 to 6 in mice fed MCD and EtOH**

Liver tissue extracts were prepared from mice fed the MCD, EtOH and ND. Prx proteins content was quantified by Western blot, using equal quantities of total liver protein. Expression levels were normalized relative to GAPDH. EtOH-fed group: drank 5% wt/vol alcohol for 2 days, 20% wt/vol alcohol for 5 days, and 30% wt/vol alcohol for 6 week.
