# Supplementary material for: Proteomic Analysis of Mice Fed Methionine and Choline Deficient Diet Reveals Marker Proteins Associated with Steatohepatitis
Source: PLoS One. 2015 Apr 7;10(4):e0120577. doi: 10.1371/journal.pone.0120577 (PMC4388516; doi:10.1371/journal.pone.0120577)
Supplement: S1 Table — (DOC) [file pone.0120577.s002.doc]

**Table S1. Methionine and Choline Deficient (MCD) Diet Composition**

| Ingredient | Amount (g/kg diet) |
| --- | --- |
| Corn starch | 100 |
| Dextrin | 100 |
| Sucrose | 408.58 |
| Cellulose | 50 |
| Corn oil | 50 |
| Salt mix #200000 | 3.5 |
| Sodium bicarbonate | 4.3 |
| Vitamin mix #300050 | 10 |
| Premix | 100 |
| Ferric citrate U.S.P | 0.12 |
| Total | 1,055.05 |
