# Supplementary material for: Proteomic Analysis of Mice Fed Methionine and Choline Deficient Diet Reveals Marker Proteins Associated with Steatohepatitis
Source: PLoS One. 2015 Apr 7;10(4):e0120577. doi: 10.1371/journal.pone.0120577 (PMC4388516; doi:10.1371/journal.pone.0120577)
Supplement: S2 Table — (DOC) [file pone.0120577.s003.doc]

**Table S2. Primers used for real time PCR**

| **Primer name** | **Sequences** |
| --- | --- |
| *GAPDH* | (F) 5′-TGG TAT CGT GGA AGG ACT CA-3′  (R) 5′-AGT GGG TGT CGC TGT TGA AG-3′ |
| *TLR4* | (F) 5′-ACC TCT GCC TTC ACT ACA GA-3′  (R) 5′-AGG GAC TTC TCA ACC TTC TC-3′ |
| *Sbp1* | (F) 5′-TCT CGC ATC TAT GTG GTG GA-3′  (R) 5′-AGT GGC TGG TGT GGA GAA AG-3′ |
| *Sbp2* | (F) 5′-AGC GCT TCT ACA AGA ATG AGG-3′  (R) 5′-GCA GCC AGT TGC TGA AGT AAA G-3′ |
| *MUP8 & 11* | (F) 5′-CAT GTT CTT GTT CTC ACA CG-3′  (R) 5′-GTC CAA TTC CAG TCT ATC C-3′ |
| *GSTP1* | (F) 5′-GTC TAC GCA GCA CTG AAT CC-3′  (R) 5′-GTA ACC ACC TCC TCC TTC CA-3′ |
| *GPX1* | (F) 5′-CAG GAG AAT GGC AAG AAT GA-3′  (R) 5′-GAA GGT AAA GAG CGG GTG AG-3′ |
| *ALDH1* | (F) 5′-TTG GAA TTT CCC GTT GGT TA-3′  (R) 5′-CTG TAG GCC CAT AAC CAG GA-3′ |
| *TNFα* | (F) 5′-AGC CCA TGT TGT AGC AAA CC-3′  (R)5′-GGA AGA CCC CTC CCA GAT AG-3′ |
| *TGFβ1* | (F) 5′-CCC AGC ATC TGC AAA GCT C-3′  (R) 5′-GTC AAT GTA CAG CTG CCG CA-3′ |
| *CD14* | (F) 5′**-**GGA AGC CAG AGA ACA CCA TC-3′  (R) 5′-CCA GAA GCA ACA GCA ACA AG**-**3′ |
